# A Recurrence-Specific Gene-Based Prognosis Prediction Model for Lung Adenocarcinoma through Machine Learning Algorithm

**DOI:** 10.1155/2020/9124792

**Published:** 2020-11-07

**Authors:** Shaohua Xu, Jie Zhou, Kai Liu, Zhoumiao Chen, Zhengfu He

**Affiliations:** ^1^Department of Thoracic Surgery, Sir Run Run Shaw Hospital, School of Medicine, Zhejiang University, 3 East Qing Chun Road, 310000 Hangzhou, Zhejiang, China; ^2^Department of Neurosurgery, Sir Run Run Shaw Hospital, School of Medicine, Zhejiang University, 3 East Qing Chun Road, 310000 Hangzhou, Zhejiang, China

## Abstract

**Background:**

After curative surgical resection, about 30-75% lung adenocarcinoma (LUAD) patients suffer from recurrence with dismal survival outcomes. Identification of patients with high risk of recurrence to impose intense therapy is urgently needed.

**Materials and Methods:**

Gene expression data of LUAD were obtained from The Cancer Genome Atlas (TCGA) and Gene Expression Omnibus (GEO) databases. Differentially expressed genes (DEGs) were calculated by comparing the recurrent and primary tissues. Prognostic genes associated with the recurrence-free survival (RFS) of LUAD patients were identified using univariate analysis. LASSO Cox regression and multivariate Cox analysis were applied to extract key genes and establish the prediction model.

**Results:**

We detected 37 DEGs between primary and recurrent LUAD tumors. Using univariate analysis, 31 DEGs were found to be significantly associated with RFS. We established the RFS prediction model including thirteen genes using the LASSO Cox regression. In the training cohort, we classified patients into high- and low-risk groups and found that patients in the high-risk group suffered from worse RFS compared to those in the low-risk group (*P* < 0.01). Concordant results were confirmed in the internal and external validation cohort. The efficiency of the prediction model was also confirmed under different clinical subgroups. The high-risk group was significantly identified as the risk factor of recurrence in LUAD by the multivariate Cox analysis (HR = 13.37, *P* = 0.01). Compared to clinicopathological features, our prediction model possessed higher accuracy to identify patients with high risk of recurrence (AUC = 96.3%). Finally, we found that the G2M checkpoint pathway was enriched both in recurrent tumors and primary tumors of high-risk patients.

**Conclusions:**

Our recurrence-specific gene-based prognostic prediction model provides extra information about the risk of recurrence in LUAD, which is conducive for clinicians to conduct individualized therapy in clinic.

## 1. Introduction

Lung cancer, the 5-year overall survival (OS) rate of which is as low as 23% [1], is the leading cancer threatening people's health worldwide [2]. Lung cancer contains two major histological types: non-small-cell lung cancer (approximately 83%) and small-cell lung cancer [1]. According to the Cancer Statistics Review 2012 [3], lung adenocarcinoma (LUAD) accounts for 43.3% of all lung cancers, replacing squamous cell lung carcinoma as the most common type of lung cancer. For early-stage LUAD patients, surgical resection is recommended [4]. However, after curative surgical resection, about 30-75% patents suffer from recurrence [5–7]. Once recurrence happens, survival outcomes are dismal, with a range of 8-14 months of postrecurrence survival (PRS) [8] and 1-year mortality as high as 48.3%-80.6% depending on the tumor stage [9, 10].

Identifying patients with high probability of submitting to recurrence and imposing intense therapy might tremendously improve the survival outcomes of LUAD. Clinical decisions for LUAD patients were mainly based on clinicopathological features like TNM stage, surgical margins, differentiation, vascular invasion, or single gene mutation status like the epidermal growth factor receptor (EGFR) mutation and the BRAF V600E mutation [11, 12]. However, these clinicopathological features fail to clearly identify patients with high risk of recurrence. Since tumorigenesis is complicated with numerous pathways regulated, researchers hypothesize that multigene profiles are capable of discriminating patients with heterogeneity survival outcomes [13]. Several groups have developed gene expression-based prediction model that successfully stratified LUAD patients into high- and low-risk groups [13–18]. Based on quantitative-PCR assay, Prof. David M Jablons developed a 14-gene expression prediction model, which stratified patients into low-risk, intermediate-risk, and high-risk groups. And the 5-year OS were 71.4%, 58.3%, and 49.2% for low-risk, intermediate-risk, and high-risk patients, respectively [19]. Benefiting from the accumulated public expression database like The Cancer Genome Atlas (TCGA) and Gene expression Omnibus (GEO), Prof. Chun-lai Lu built a risk model by screening prognostic-related genes using expression data from TCGA [20]. Many of these gene signatures are based on literature review or roughly screening survival-related genes using the Cox regression, which makes them not stable enough to be generalized in clinic.

It is rationally hypothesized that building a prediction model on the basis of recurrence-specific genes would better distinguish high-risk patients of recurrence. Therefore, aiming to identify high-risk LUAD patients of recurrence, we explored the recurrence-associated genes using the public GEO dataset and established a recurrence-free survival (RFS) prediction model using the expression data of LUAD patients from TCGA and validated its accuracy and feasibility in an external dataset.

## 2. Methods

### 2.1. Dataset Description

Gene expression profiles of primary and recurrent LUAD (GSE7880) and the external validation cohort (GSE68465) were downloaded from the Gene Expression Omnibus (GEO) website (https://www.ncbi.nlm. http://nih.gov/geo). The expression matrix of The Cancer Genome Atlas (TCGA) cohort was downloaded from the TCGA website (https://xenabrowser.net/datapages/), along with the matched clinical records. Patients without clear RFS were filtered out. Patients with sufficient RFS obtained from the TCGA database were randomly divided into the training and testing subsets, with a ratio of 7 : 3.

### 2.2. Identification of Differentially Expressed Genes (DEGs)

The differentially expressed genes (DEGs) of the microarray-based data (GSE7880) were identified using the “limma” package [21] while the DEGs of sequencing-based data (TCGA) were identified using the “DESeq2” package [22]. DEGs of both datasets were determined based on an absolute log2 (fold change) > 1 and a *P* value less than 0.05. The GSEA [23] software was used to calculate the normalized enrichment scores (NES) and false discovery rate (FDR) values for the Hallmark gene sets [24]. The genes were preranked according to the log fold change values. NES corresponds to the enrichment score (ES), which reflects the degree to which a gene set is overrepresented at the top or bottom of a ranked list of genes. The normalization is based on the gene set enrichment scores for all dataset permutations.

### 2.3. Development of Prediction Model

All data analyses were calculated using the related R packages on the R platform (https://cran.r-project.org/src/base/R-3/) (version 3.6.2). The univariate and multivariate Cox regression analyses were carried out using the “survival” package (v3.1-8). We normalized TCGA gene expression value into log_2_ (TPM + 1) and performed the univariate Cox regression analysis to find out the RFS-related gene candidates (*P* < 0.05) using the DEGs (note: TPM, transcripts per kilobase of exon model per million mapped reads). Then, the LASSO Cox regression analysis was carried out to select features (gene signature) with the best prediction power. The multivariate Cox regression analysis was performed to construct the prognostic model using the selected features, by which we calculated the risk scores of each patient and separated them into low- (risk score < 0) and high-risk (risk score > 0) subgroups. The survival plot was calculated with the “rms” package (v5.1-4), which were used to detect the significant difference of RFS risks between these two subgroups, and the logrank test was performed to state the differential significance between the two subgroups. Besides, the receiver operating characteristic (ROC) curve was employed to test the stability and sensitivity of this prognostic model using the R package “pROC” (v1.16.1) [25].

## 3. Results

### 3.1. Identification of LUAD Recurrence Specific Genes

**Figure 1:**
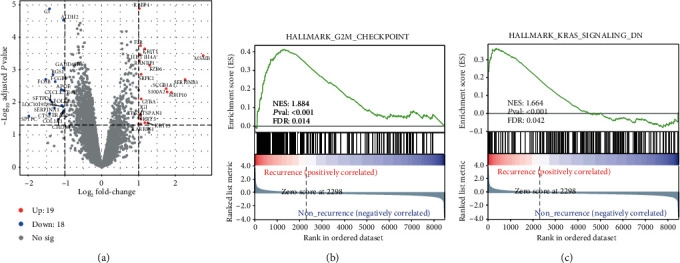
Differentially expressed genes between recurrent and primary LUAD: (a) the volcano plot displaying DEGs between recurrent and primary LUAD samples in the GSE7880 cohort; (b, c) bar plot showing the G2M checkpoint pathway (b) and KRAS signaling pathway (c) enriched in recurrent tumors using the gene set enrichment analysis.

Aiming to identify genes associated with LUAD recurrence, we collected an expression microarray dataset containing primary and recurrent LUAD samples from GEO (GSE7880). We detected the DEGs between primary and recurrent LUAD using the “limma” package. Genes with absolute log2 (fold change) > 1 and *P* value < 0.05 were considered statistically significant DEGs. In all, we identified 37 DEGs, including 19 upregulated genes and 18 downregulated genes in recurrent tumors (Figure 1(a); Table S1). Gene set enrichment analysis (GSEA) indicated that recurrent LUAD was associated with the activity of the G2M checkpoint pathway (NES = 1.88; FDR = 0.01) and KRAS signaling pathway (NES = 1.66; FDR = 0.04) (Figures 1(b) and 1(c)).

### 3.2. Establishment of Recurrence-Specific Gene-Based RFS Predicting Model

**Figure 2:**
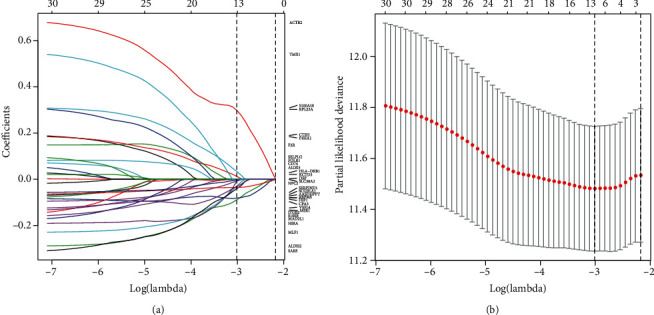
Development of recurrence-specific gene-based RFS predicting model. (a) Coefficient profile plot was produced against the log lambda sequence. (b) Tuning parameter (lambda) selection in the LASSO model used 10-fold cross-validation via minimum criteria.

In order to develop a robust RFS predicting model for LUAD, we collected the expression data of 426 LUAD patients from TCGA with available clear RFS. We extracted the 37 DEGs' expression profile from TCGA datasets and performed the univariate Cox regression analysis to identify RFS-related gene candidates. As a result, 31 genes were found significantly associated with the RFS of LUAD patients (*P* < 0.05, logrank test; Figure S1). Then, we randomly selected 70% of patients from TCGA dataset as the training cohort (298 samples) and the rest as testing cohort (128 samples) (Table 1). The LASSO Cox regression analysis was applied to extract key genes with most RFS prediction power in training cohort. Finally, thirteen key genes including *ACTR2*, *ALDH2*, *FBP1*, *HIRA*, *ITGB2*, *MLF1*, *P4HA1*, *S100A10*, *S100B*, *SARS*, *SCGB1A1*, *SERPIND1*, and *VSIG4* were extracted to establish the RFS prediction model. We established a RFS predicting model referring to the gene expression using the multivariate Cox regression: Risk score = 0.469 × expression (*ACTR*2) − 0.210 × expression (*ALDH*2) − 0.081 × expression (*FBP*1) − 0.328 × expression (*HIRA*) + 0.012 × expression (*ITGB*2) − 0.203 × expression (*MLF*1) + 0.135 × expression (*P*4*HA*1) + 0.181 × expression (*S*100*A*10) − 0.074 × expression (*S*100*B*) − 0.189 × expression (*SARS*) − 0.044 × expression (*SCGB*1*A*1) − 0.050 × expression (*SERPIND*1) − 0.137 × expression (*VSIG*4) (Figure 2). Risk score < 0 infers patients with low risk of recurrence, while risk score > 0 infers patients with high risk of recurrence.

### 3.3. Efficiency of the RFS Prediction Model

**Figure 3:**
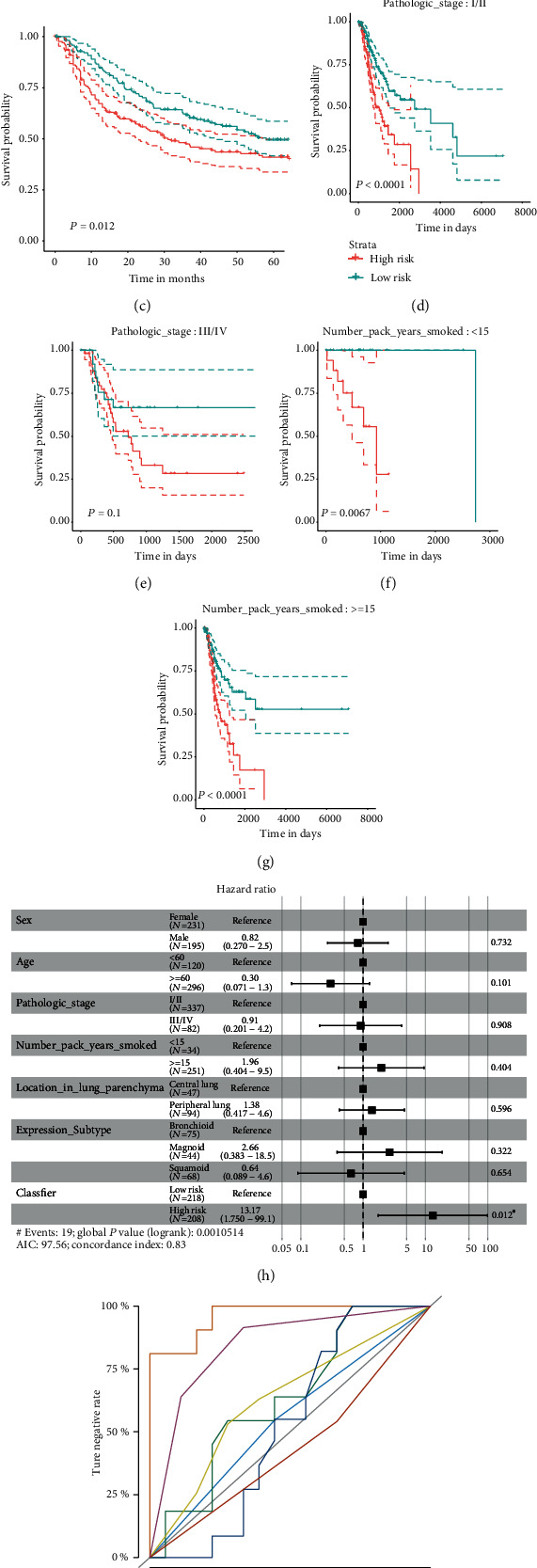
Efficiency of RFS prediction model. (a) The Kaplan-Meier (K-M) curve confirmed that the signature could significantly distinguish low- and high-risk groups in the training cohort. (b) The K-M curve confirmed that the signature could significantly distinguish low- and high-risk groups in the internal validation cohort. (c) The K-M curve confirmed that the signature could significantly distinguish low- and high-risk groups in the external validation cohort (GSE68465). (d–g) The K-M curve confirmed that the prediction model could distinguish low- and high-risk groups in the pathological subgroups (d, e) and smoking history subgroups (f, g). (h) Forest plot showed results of multivariate cox analysis. (i) Receiver operating characteristic curve showed the prediction model obtained good predictive effect compared to other clinicopathological features.

Using the RFS prediction model, 48.66% and 49.22% of patients were classified into the high-risk group in the training and validation cohort, respectively. We found that patients with high risk suffered from worse RFS compared to patients with low risk in the training cohort (median RFS: 795 days vs. 3521 days; *P* < 0.01, logrank test; Figure 3(a)). Concordantly, similar result was further confirmed in the validation cohort (median RFS: 1084 days vs. 2701 days; *P* = 0.03, logrank test; Figure 3(b)). Furthermore, we validated the efficiency of the prediction model using an external validation cohort (443 patients) reported by Prof. David G Beer [26] from the GEO database (GSE68465; Table 1). After extracting the expression data of thirteen key genes, we categorize patients into high-risk and low-risk groups as previously elaborated. As expected, patients with high risk suffered from worse RFS compared to patients with low risk (median RFS: 31.50 months vs. 59.17 months; *P* = 0.01, logrank test; Figure 3(c)). Furthermore, we evaluated the efficiency of our prediction model in clinicopathological subgroups. In subgroup of age, gender, pathologic stage, smoking history, and location in lung parenchyma, better RFS happened in patients of the low-risk group compared to those of the high-risk group (Figures 3(d)–3(g); Figure S2A–F).

Combining the clinicopathological features including patient age, gender, pathologic stage, smoking history, location in lung parenchyma, and expression subtype [27] with our prediction signature, we performed the multivariate Cox regression analysis. Except our prediction signature, none of the clinicopathological signatures was related to the risk of RFS (HR = 13.37, CI: 1.75-99.10, *P* = 0.01, logrank test; Figure 3(h)). Also, we wonder whether the efficiency of the DEG-based signature is better than other clinicopathological signatures, so we compared the stability and sensitivity of the RFS prediction model using the ROC curve (Figure 3(i)). Compared to other clinicopathological features, the RFS prediction model achieved the supreme efficiency of predicting RFS (AUC = 96.3%; Figure 3(i)). Taken together, the recurrence-specific gene-based signature is capable of better stratifying LUAD patients into high- and low-risk groups compared to other clinicopathological features.

### 3.4. Key Pathways Associated with the High Risk of Recurrence in LUAD

**Figure 4:**
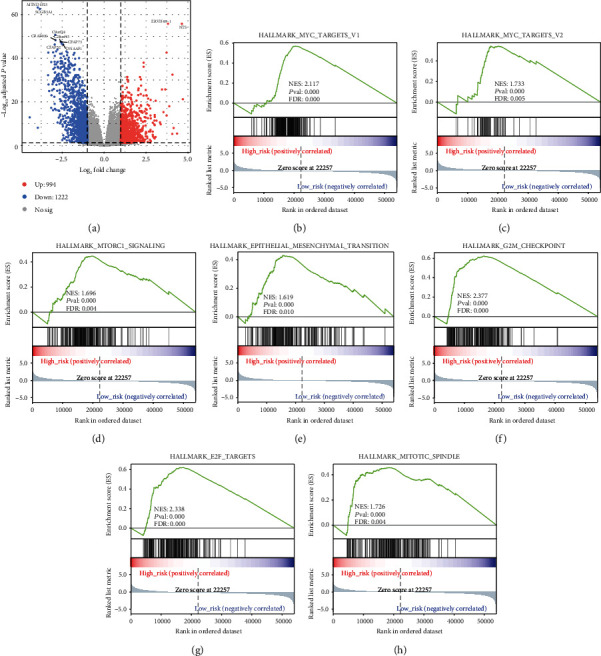
Key pathways associated with high risk of recurrence in LUAD. (a) The volcano plot displaying DEGs between high- and low-risk LUAD in the entire TCGA cohort. (b–h) Gene set enrichment analysis shows the Hallmark pathways enriched in high-risk patients.

In order to figure out the key molecular pathways associated with the recurrence of LUAD, we detected the DEGs between patients with high risk and those with low risk in the entire TCGA LUAD cohort. In all, we detected 2216 significant DEGs, which consist of 994 upregulated genes and 1222 downregulated genes in the high-risk group (Figure 4(a); Table S3). Then, GSEA found that the MYC target pathway (NES = 2.12; FDR < 0.01), mTORC1 signaling pathway (NES = 1.69; FDR = 0.004), epithelial mesenchymal transition (EMT) pathway (NES = 1.62; FDR = 0.01), and cell cycle-related pathway like the G2M checkpoint pathway (NES = 2.377; FDR < 0.01), E2F target pathway (NES = 2.33; FDR < 0.01), and mitotic spindle pathway (NES = 1.72; FDR < 0.01) were enriched in high-risk patients (Figures 4(b)–4(h)). As previously reported, all these pathways were associated with tumor progression [28–33]. It is noted that the G2M checkpoint pathway was the only pathway that was enriched in both recurrent tumors and primary tumor with high risk of recurrence (Figures 1(b) and 4(f)), which indicates its potential as treatment targets for patients prone to recurrence.

## 4. Discussion

With the aim of identifying LUAD patients with heterogeneity RFS, we detected the DEGs between primary and recurrent LUAD tumors, extracted RFS-associated genes, and established the RFS prediction model using a machine learning algorithm based on a large cohort. Using the prediction model, we classified the patients into high- and low-risk groups and found that patients in the high-risk group suffered from worse RFS compared to those in the low-risk group. Concordant results were confirmed in the internal and external validation cohort. Compared to clinicopathological features, our prediction model possessed higher accuracy to identify patients with high risk of recurrence. Finally, we found that the G2M checkpoint pathway was enriched in both recurrent tumors and primary tumors of high-risk patients.

Due to the high proportion of recurrence that occurred in LUAD, more and more researchers realize the importance of identification of patients with high risk of recurrence. Considering the limitations of clinicopathological features, combination of multisurvival-associated genes might be an ideal way to solve this problem, and pilot studies achieved significant progress [13–15, 19, 20, 26]. Instead of extracting genes merely associated with survival outcomes using the Cox regression analysis, we developed our prediction model based on recurrence-specific genes using the machine learning algorithm. Most genes included in our final prediction model were reported to be related to survival in lung cancer or other cancers [34–42], which indicates the rationality of our prediction model. For example, high expression of *P4HA1* and *S100A10* was reported to be associated with dismal survival outcomes in LUAD [36, 38]. Prof. Xiao-jing Wang found that *MLF1* promotes the proliferation and colony-forming abilities of lung adenocarcinoma cells and significantly decreases apoptosis in vitro [39]. Since we reported the conduction of our prediction model clearly, it is feasible and convenient for clinicians to design the specific target panel and apply it in clinic to evaluate the risk of recurrence for each patient. Our prediction model provides extra information about the risk of recurrence, which is conducive for clinicians to identify high-risk patients and impose intense therapy like adjuvant chemotherapy.

The G2M checkpoint pathway was found to be enriched both in recurrent tumors and primary tumors of high-risk patients, which infers its important association with recurrence. G2M checkpoint is an essential process of cell cycle which ensures that cells do not initiate mitosis until damaged or incompletely replicated DNA is sufficiently repaired. Thus, cell cycle checkpoint is the critical barrier to preserve genome integrity and chromosomal stability and prevent progression of tumors from early stages to malignant invasive lesions [29]. Expression of genes involved in cell cycle checkpoint pathway has been reported to be related to the survival outcomes in lung cancer [29, 43]. For example, overexpression of PLK1, an early trigger for G2/M transition, is a negative prognostic factor in non-small-cell lung cancer patients [44]. Due to its critical role in tumorigenesis and progression, inhibitors of cell cycle regulators have attracted intense research interests [29]. As an example, MK-0457 (VX-680) blocks tumor xenograft growth and induces tumor regression in preclinical models [45]. Since the G2M checkpoint pathway was significantly enriched in recurrent and high-risk patients, combination of inhibitors of cell cycle regulators and traditional chemotherapy or radiotherapy might achieve improved efficacy in patients with high risk of recurrence.

In conclusion, the signature we developed using the recurrence-specific genes is robust in predicting RFS outcomes of LUAD. Our prediction model provides extra information about the risk of recurrence, which is conducive for clinicians to conduct individualized therapy in clinic. To further apply in clinic, multicenter-based large-scale studies are warranted to verify the feasibility and stability of the model.

## Figures and Tables

**Table 1:** Clinical characteristics of included patients for survival model construction and validation.

	TCGA training cohort (288)	TCGA testing cohort (128)	External validation cohort (335)
Sex		*P* = 0.30	*P* = 0.99
Female	167 (56.04%)	64 (50%)	189 (56.42%)
Male	131 (43.96%)	64 (50%)	146 (43.58%)
Age		*P* = 0.33	*P* = 1.00
≥60	201 (67.45%)	95 (74.22%)	234 (69.85%)
<60	88 (29.53%)	32 (25%)	101 (30.15%)
Unknown	9 (3.02%)	1 (0.78%)	0 (0%)
Pathologic T		*P* = 0.27	*P* = 0.32
T1	109 (36.58%)	41 (32.03%)	110 (32.84%)
T2	160 (53.69%)	67 (52.34%)	202 (60.29%)
T3	21 (7.05%)	13 (10.16%)	16 (4.78%)
T4	6 (2.01%)	6 (4.69%)	5 (1.49%)
Unknown	2 (0.67%)	1 (0.78%)	2 (0.60%)
Pathologic N		*P* = 0.66	*P* = 0.31
N0	201 (67.45%)	80 (62.50%)	299 (89.25%)
N1	52 (17.45%)	26 (20.31%)	88 (26.27%)
N2	38 (12.75%)	17 (13.28%)	53 (14.93%)
N3	2 (0.67%)	0 (0%)	0 (0%)
Unknown	5 (1.68%)	5 (3.91%)	0 (0%)
Pathologic M		*P* = 1.00	NA
M0	192 (64.43%)	83 (64.84%)	0 (0%)
M1	12 (4.03%)	5 (3.91%)	0 (0%)
Unknown	94 (31.54%)	40 (31.25%)	335 (100%)
Tumor stage		*P* = 0.70	*P* < 0.01
I	171 (57.38%)	64 (50.00%)	150 (33.86%)
II	69 (23.15%)	33 (25.78%)	252 (56.88%)
III	43 (14.43%)	21 (16.41%)	29 (6.55%)
IV	12 (4.03%)	6 (4.69%)	12 (2.71%)
Unknown	3 (1.01%)	4 (3.13%)	0 (0%)

## Data Availability

The data used to support the findings of this study are included within the article. The data and materials in the current study are available from the corresponding author on reasonable request.

## Supplementary Materials

Supplementary Materials Figure S1: univariate Cox regression analysis to screen out the recurrence-free survival-related differentially expressed genes. Figure S2: efficiency of prediction model under different clinical subgroups. The K-M curve confirmed that the signature could significantly distinguish low- and high-risk groups in the sex subgroups (A and B), age subgroups (C and D), and location in lung parenchyma subgroups (E and F). Table S1: differential expressed genes between recurrent and primary LUAD. Table S2: differential expressed genes between high and low risks of LUAD patients of TCGA.
