# Supplementary material for: A Recurrence-Specific Gene-Based Prognosis Prediction Model for Lung Adenocarcinoma through Machine Learning Algorithm
Source: Biomed Res Int. 2020 Nov 7;2020:9124792. doi: 10.1155/2020/9124792 (PMC7669350; doi:10.1155/2020/9124792)
Supplement: Supplementary Materials — Figure S1: univariate Cox regression analysis to screen out the recurrence-free survival-related differentially expressed genes. Figure S2: efficiency of prediction model under different clinical subgroups. The K-M curve confirmed that the signature could significantly distinguish low- and high-risk groups in the sex subgroups (A and B), age subgroups (C and D), and location in lung parenchyma subgroups (E and F). Table S1: differential expressed genes between recurrent and primary LUAD. Table S2: differential expressed genes between high and low risks of LUAD patients of TCGA. [file 9124792.f1.zip › Fractional HBV model_diffusionR1.pdf]

Analysis of a fractional reaction-diffusion HBV model with cure of infected cells

Moussa Bachraoui [a], **Khalid Hattaf** [a,b], **Noura Yousfi**[a]

[a]Laboratory of Analysis, Modeling and Simulation (LAMS), Faculty of Sciences Ben M'sik, Hassan II University of Casablanca, P.O Box 7955 Sidi Othman, Casablanca, Morocco
[b]Centre Régional des Métiers de l'Education et de la Formation (CRMEF), 20340 Derb Ghalef, Casablanca, Morocco

Abstract

In this paper, we propose a fractional reaction-diffusion model in order to better understand the mechanisms and dynamics of hepatitis B virus (HBV) infection in human body. The infection transmission is modeled by Hattaf-Yousfi functional response and the fractional derivative is in the sense of Caputo. The global stability of the model equilibria is analyzed by means of Lyapunov functionals. Finally, numerical simulations are presented to support our analytical results.

Keywords: HBV infection, diffusion, fractional partial differential equations, global asymptotic stability.

1 Introduction

In the recent years, fractional calculus has attracted the attention of many researchers. Hattaf [1] proposed a new fractional derivative with non-singular kernel which generalizes many forms existing in the literature such as the Caputo-Fabrizio and Atangana-Baleanu fractional derivatives. Further, there are some new methods used to solve numerically fractional models considered to explain deeper investigations of real world problems [2–6].

On the other hand, hepatitis B is a dangerous infectious disease caused by the hepatitis B virus (HBV). It affects the lives of 257 million people and responsible for the deaths of 56000 people every year according to World Health Organization (WHO) estimates [7]. Therefore, several mathematical models have been proposed and developed to describe the dynamics of HBV

dynamics. For instance, Manna and Chakrabarty [8] proposed and analyzed the dynamics of HBV infection by taking into account the spacial mobility of both HBV DNA-containing capsids and viruses. Their work was an extension of that presented in [9]. Guo et al. [10] studied a nonlinear system of partial differential equations (PDEs) for HBV infection with three time delays, general incidence rate and spatial diffusion only in the viruses. Hattaf and Yousfi [11] developed a mathematical HBV infection model with two modes of transmission, and allows the movement of HBV DNA-containing capsids and viruses, and three distributed delays. Since fractional order models possess property of memory, Bachraoui et al. [12] proposed a mathematical model governed by fractional differential equations (FDEs) to more explore the dynamic characteristics of the HBV infection. They have improved and generalized the ordinary differential equation (ODE) models [9, 13] and also the FDE models [14–16] by using the Hattaf's incidence rate [17] that includes the common types such as the bilinear incidence rate, the saturated incidence rate and the Beddington-DeAngelis functional response [18, 19].

In this study, we present an extension of our model presented in [12] by considering the mobility of capsids and viruses. So, we propose the following mathematical model formulated by fractional partial differential equations (FPDEs) to better describe the dynamics of HBV infection under the effects of diffusion and memory:

$$\begin{cases} \partial_t^\alpha U &= \sigma - \delta U(x,t) - F(U(x,t),V(x,t))V(x,t) + \epsilon I(x,t), \\ \partial_t^\alpha I &= F(U(x,t),V(x,t))V(x,t) - (\rho+\epsilon)I(x,t), \\ \partial_t^\alpha C &= d_C \Delta C + \kappa I(x,t) - (\rho+\eta)C(x,t), \\ \partial_t^\alpha V &= d_V \Delta V + \eta C(x,t) - \nu V(x,t). \end{cases} \tag{1}$$

The state variables $U(x,t)$, $I(x,t)$, $C(x,t)$ and $V(x,t)$ are respectively the concentrations of uninfected liver cells, infected liver cells, HBV DNA-containing capsids and virions at location x and time t. Uninfected liver cells are produced at constant rate σ, die at rate δU and become infected by virus at rate $F(U,V)V$. The parameters ϵ, ρ κ, η and ν are respectively the cure rate of infected liver cells, the death rate of infected liver cells and capsids, the production rate of capsids from infected liver cells, the rate at which the capsids are converted to virions, and the viral clearance rate. The positive constants d_C and d_V are the diffusion coefficients of capsids and virus. $\Delta = \sum_{i=1}^{n} \frac{\partial^2}{\partial x_i^2}$ is

the Laplacian operator. The incidence function of (1) is described by Hattaf-Yousfi functional response [17] of the form $F(U,V) = \frac{bU}{\alpha_0+\alpha_1 U+\alpha_2 V+\alpha_3 UV}$, where the nonnegative constants α_i, $i = 0,1,2,3$, measure the saturation, inhibitory or psychological effects, and the positive constant b is the infection rate. This functional response covers several specific cases available in the literature such as the bilinear and saturation incidences, the Beddington-DeAnglis and Crowley-Martin functional responses, and the specific functional response introduced by Hattaf et al. [20]. Finally, ∂_t^α is the Caputo fractional derivative of order $\alpha \in (0,1]$. The choice of this type of fractional derivative is motivated by the fact that the fractional derivative of a constant is equal to zero, and α was chosen in the interval $(0,1]$ to have the same initial conditions as those of the PDE systems. Further, a recent study in [21] has shown that the fractional order model gives better predictions than that of the integer model about the plasma virus load of the patients.

Throughout this paper, we consider system (1) with initial conditions

$$\begin{aligned} U(x,0) &= U_0(x) \geq 0,\ I(x,0) = I_0(x) \geq 0, \\ V(x,0) &= V_0(x) \geq 0,\ C(x,0) = C_0(x) \geq 0,\ \forall x \in \bar{\Omega}, \end{aligned} \tag{2}$$

and zero-flux boundary conditions

$$\frac{\partial C}{\partial \vec{n}} = \frac{\partial V}{\partial \vec{n}} = 0, \quad \text{on } \partial\Omega \times (0,+\infty),$$

where Ω is a bounded domain in $\mathbb{R}^n$ with smooth boundary $\partial\Omega$, and $\frac{\partial}{\partial \vec{n}}$ denotes the outward normal derivative on $\partial\Omega$. From the biological point of view, these boundary conditions indicate that the capsids and free virus particles do not move across the boundary $\partial\Omega$.

The rest of the paper is organized as follows. The following section is devoted to the calculations of the basic reproduction number and steady states of model (1). The global dynamics of the FPDE model is analyzed in Section 3. To support the analytical results, we present some numerical simulations in Section 4. Finally, we end the paper with biological and mathematical conclusions in Section 5.

2 Equilibria of the FPDE model

It is easy to verify that the only infection-free steady state of the FPDE model (1) is $P_0(U^0,0,0,0)$, where $U^0 = \dfrac{\sigma}{\delta}$. Then the basic reproduction

number of (1) given by

$$\mathcal{R}_0 = \frac{\kappa\eta F\left(U^0, 0\right)}{\nu(\eta+\rho)(\epsilon+\rho)}. \tag{3}$$

The other steady states verify the following system:

$$\begin{aligned}
\sigma - \delta U - F(U,V)V + \epsilon I &= 0, && (4)\\
F(U,V)V - (\epsilon+\rho)\, I &= 0, && (5)\\
\kappa I - (\eta+\rho)C &= 0, && (6)\\
\eta C - \nu V &= 0. && (7)
\end{aligned}$$

From (4)-(7), we get $I = \frac{\sigma-\delta U}{\rho}$, $C = \frac{\kappa(\sigma-\delta U)}{\rho(\eta+\rho)}$, $V = \frac{\eta\kappa(\sigma-\delta U)}{\nu\rho(\eta+\rho)}$ and

$$F\left(U, \frac{\eta\kappa(\sigma-\delta U)}{\nu\rho(\eta+\rho)}\right) = \frac{\nu(\eta+\rho)\,(\rho+\epsilon)}{\kappa\eta}. \tag{8}$$

$I = \frac{\sigma-\delta U}{\rho} \geqslant 0$ implies that $U \leqslant \dfrac{\sigma}{\delta}$. So, we consider the function G defined on interval $[0, \dfrac{\sigma}{\delta}]$ by

$$G(U) = F\left(U, \frac{\eta\kappa(\sigma-\delta U)}{\nu\rho(\eta+\rho)}\right) - \frac{\nu(\eta+\rho)\,(\rho+\epsilon)}{\kappa\eta}. \tag{9}$$

We have $G(0) = -\frac{\nu(\eta+\rho)(\rho+\epsilon)}{\kappa\eta} < 0$, $G\left(\dfrac{\sigma}{\delta}\right) = \frac{\nu(\eta+\rho)(\rho+\epsilon)}{\kappa\eta}\left(\mathcal{R}_0 - 1\right)$ and

$$G'(U) = \frac{\partial F}{\partial U} - \frac{\kappa\eta\delta}{\nu\rho(\eta+\rho)}\frac{\partial F}{\partial V} > 0. \tag{10}$$

If $\mathcal{R}_0 > 1$, we deduce that system (1) admits a unique infection equilibrium $P_1(U_1, I_1, C_1, V_1)$ with $U_1 \in \left(0, \dfrac{\sigma}{\delta}\right)$, $I_1 = \frac{\sigma-\delta U_1}{\rho}$, $C_1 = \frac{\kappa(\sigma-\delta U_1)}{\rho(\eta+\rho)}$ and $V_1 = \frac{\eta\kappa(\sigma-\delta U_1)}{\nu\rho(\eta+\rho)}$.

We summarize the above discussions in the following result.

Theorem 2.1.

(i) When $\mathcal{R}_0 \leq 1$, the FPDE model (1) has one infection-free steady state $P_0(U^0, 0, 0, 0)$, where $U^0 = \dfrac{\sigma}{\delta}$.

(ii) When $\mathcal{R}_0 > 1$, the FPDE model (1) has uniquely one chronic infection steady state $P_1(U_1, I_1, C_1, V_1)$, where $U_1 \in \left(0, \dfrac{\sigma}{\delta}\right)$, $I_1 = \frac{\sigma-\delta U_1}{\rho}$, $C_1 = \frac{\kappa(\sigma-\delta U_1)}{\rho(\eta+\rho)}$ and $V_1 = \frac{\eta\kappa(\sigma-\delta U_1)}{\nu\rho(\eta+\rho)}$.

3 Global dynamics

This section analyzes the global dynamics of the FPDE model (1).

Theorem 3.1. *The infection-free steady state P_0 is globally asymptotically stable if $\mathcal{R}_0 \leq 1$.*

Proof. Let

$$\begin{aligned} L_0(t) &= \int_{\Omega} \left[\frac{\alpha_0}{\alpha_0+\alpha_1 U^0} U^0 \Phi\left(\frac{U}{U^0}\right) + \frac{\alpha_0 \epsilon \left(U(x,t) - U^0 + I(x,t)\right)^2}{2\left(\rho+\delta\right)\left(\alpha_0+\alpha_1 U^0\right) U^0} \right. \\ &\quad \left. + I(x,t) + \frac{\epsilon+\rho}{\kappa} C(x,t) + \frac{(\epsilon+\rho)\left(\eta+\rho\right)}{\kappa\eta} V(x,t) \right] dx, \end{aligned}$$

where $\Phi(x) = x - 1 - \ln(x)$ for $x > 0$. According to [22], we obtain

$$\begin{aligned} D^{\alpha} L_0(t) &\leq \int_{\Omega} \left[\frac{\alpha_0}{\alpha_0+\alpha_1 U^0} \left(1 - \frac{U^0}{U}\right) \partial_t^{\alpha} U + \partial_t^{\alpha} I + \frac{\alpha_0 \epsilon \left(U - U^0 + I\right)\left(\partial_t^{\alpha} U + \partial_t^{\alpha} I\right)}{\left(\rho+\delta\right)\left(\alpha_0+\alpha_1 U^0\right) U^0} \right. \\ &\quad \left. \frac{\epsilon+\rho}{\kappa} \partial_t^{\alpha} C + \frac{(\epsilon+\rho)\left(\eta+\rho\right)}{\kappa\eta} \partial_t^{\alpha} V \right] dx. \end{aligned}$$

By $\sigma = \delta U^0$, we have

$$\begin{aligned} D^{\alpha} L_0(t) &\leq \int_{\Omega} \left[-\left(\frac{1}{U} + \frac{\epsilon}{(\rho+\delta) U^0}\right) \frac{\alpha_0 \delta (U-U^0)^2}{\alpha_0+\alpha_1 U^0} - \frac{\alpha_0 \epsilon \rho I^2}{\left(\rho+\delta\right)\left(\alpha_0+\alpha_1 U^0\right) U^0} \right. \\ &\quad \left. - \frac{\alpha_0 \epsilon I \left(U-U^0\right)^2}{(\alpha_0+\alpha_1 U^0) U^0 U} + \frac{\nu(\epsilon+\rho)(\eta+\rho)}{\kappa\eta} \left(\mathcal{R}_0 \frac{F(U,V)}{F(U^0,0)} - 1\right) V \right] dx, \\ &\leq -\int_{\Omega} \left[\left(\frac{1}{U} + \frac{\epsilon}{(\rho+\delta) U^0}\right) \frac{\alpha_0 \delta (U-U^0)^2}{\alpha_0+\alpha_1 U^0} + \frac{\alpha_0 \epsilon \rho I^2}{\left(\rho+\delta\right)\left(\alpha_0+\alpha_1 U^0\right) U^0} \right. \\ &\quad \left. + \frac{\alpha_0 \epsilon I \left(U-U^0\right)^2}{(\alpha_0+\alpha_1 U^0) U^0 U} - \frac{\nu(\epsilon+\rho)(\eta+\rho)}{\kappa\eta} \left(\mathcal{R}_0 - 1\right) V \right] dx. \end{aligned}$$

Then $D^{\alpha} L_0(t) \leq 0$ when $\mathcal{R}_0 \leq 1$. Additionally, $\{P_0\}$ is the largest invariant set in $\{(U, I, C, V) \mid D^{\alpha} L_0(t) = 0\}$. By LaSalle's invariance principale [23], P_0 is globally asymptotically stable if $\mathcal{R}_0 \leq 1$. ■

Theorem 3.2. *The chronic infection steady state P_1 is globally asymptotically stable when $\mathcal{R}_0 > 1$ and*

$$\mathcal{R}_0 \leq 1 + \frac{\left[\delta\rho\nu(\eta+\rho) + \alpha_2 \delta\sigma\kappa\eta\right](\epsilon+\rho) + \alpha_3 \epsilon\eta\kappa\sigma^2}{\epsilon\nu\rho(\epsilon+\rho)(\alpha_0\delta + \alpha_1\sigma)}. \tag{11}$$

Proof. Let

$$\begin{aligned} L_1(t) &= \int_\Omega \left[\frac{\alpha_0+\alpha_2V_1}{\alpha_0+\alpha_1U_1+\alpha_2V_1+\alpha_3U_1V_1} U_1\Phi\left(\frac{U}{U_1}\right) \right. \\ &\quad + \frac{\epsilon\left(\alpha_0+\alpha_2V_1\right)\left(U(x,t)-U_1+I(x,t)-I_1\right)^2}{2\left(\rho+\delta\right)\left(\alpha_0+\alpha_1U_1+\alpha_2V_1+\alpha_3U_1V_1\right)U_1} \\ &\quad \left. + I_1\Phi\left(\frac{I}{I_1}\right) + \frac{\epsilon+\rho}{\kappa}C_1\Phi\left(\frac{C}{C_1}\right) + \frac{\left(\epsilon+\rho\right)\left(\eta+\rho\right)}{\kappa\eta}V_1\Phi\left(\frac{V}{V_1}\right)\right] dx. \end{aligned}$$

Then

$$\begin{aligned} D^\alpha L_1(t) &\leq \int_\Omega \left[\frac{\alpha_0+\alpha_2V_1}{\alpha_0+\alpha_1U_1+\alpha_2V_1+\alpha_3U_1V_1}\left(1-\frac{U_1}{U}\right)\partial_t^\alpha U \right. \\ &\quad + \frac{\epsilon\left(\alpha_0+\alpha_2V_1\right)\left(U-U_1+I-I_1\right)\left(\partial_t^\alpha U+\partial_t^\alpha I\right)}{\left(\rho+\delta\right)\left(\alpha_0+\alpha_1U_1+\alpha_2V_1+\alpha_3U_1V_1\right)U_1} + \left(1-\frac{I_1}{I}\right)\partial_t^\alpha I \\ &\quad \left. + \frac{\epsilon+\rho}{\kappa}\left(1-\frac{C_1}{C}\right)\partial_t^\alpha C + \frac{\left(\epsilon+\rho\right)\left(\eta+\rho\right)}{\kappa\eta}\left(1-\frac{V_1}{V}\right)\partial_t^\alpha V\right] dx. \end{aligned}$$

Since $\sigma = \delta U_1 + \rho I_1,\ F(U_1,V_1)V_1 = (\epsilon+\rho)I_1,\ \kappa I_1 = (\eta+\rho)C_1$ and $1 - \dfrac{F(U_1,V_1)}{F(U,V_1)} = \dfrac{\alpha_0+\alpha_2V_1}{\alpha_0+\alpha_1U_1+\alpha_2V_1+\alpha_3U_1V_1}\left(1-\dfrac{U_1}{U}\right)$, we have

$$\begin{aligned} D^\alpha L_1(t) &\leq \int_\Omega \left[\frac{-\delta\left(\alpha_0+\alpha_2V_1\right)\left(U-U_1\right)^2}{\left(\alpha_0+\alpha_1U_1+\alpha_2V_1+\alpha_3U_1V_1\right)U} + \frac{\epsilon\left(\alpha_0+\alpha_2V_1\right)\left(U-U_1\right)\left(I-I_1\right)}{\left(\alpha_0+\alpha_1U_1+\alpha_2V_1+\alpha_3U_1V_1\right)U} \right. \\ &\quad + F(U,V_1)V_1\left(1-\frac{F(U_1,V_1)}{F(U,V_1)}+\frac{F(U,V)V_1}{F(U,V_1)V_1}\right) + F(U_1,V_1)V_1\left(1-\frac{IC_1}{I_1C}\right) \\ &\quad + F(U_1,V_1)V_1\left(1-\frac{F(U,V)VI_1}{F(U_1,V_1)IV_1}\right) + F(U_1,V_1)V_1\left(1-\frac{V}{V_1}-\frac{CV_1}{C_1V}\right) \\ &\quad \left. - \frac{\epsilon\left(\alpha_0+\alpha_2V_1\right)\left[\delta\left(U-U_1\right)^2+\rho(I-I_1)^2+\left(\delta+\rho\right)\left(U-U_1\right)\left(I-I_1\right)\right]}{\left(\alpha_0+\alpha_1U_1+\alpha_2V_1+\alpha_3U_1V_1\right)\left(\delta+\rho\right)U_1}\right] dx \\ &\quad - \frac{\epsilon+\rho}{\kappa}d_C C_1\int_\Omega \frac{\left\|\triangledown C\right\|^2}{C^2}dx - \frac{\left(\epsilon+\rho\right)\left(\eta+\rho\right)}{\kappa\eta}d_V V_1 \int_\Omega \frac{\left\|\triangledown V\right\|^2}{V^2}dx. \end{aligned}$$

Hence,

$$
\begin{aligned}
D^{\alpha}L_1(t) \quad \leq \quad & -\int_{\Omega}\left[\frac{(\alpha_0+\alpha_2V_1)(U-U_1)^2}{(\alpha_0+\alpha_1U_1+\alpha_2V_1+\alpha_3U_1V_1)\,UU_1}\left((\delta U_1-\epsilon I_1)+\frac{\delta\epsilon U}{\rho+\delta}+\epsilon I\right)\right. \\
& +\frac{\epsilon\rho\,(\alpha_0+\alpha_2V_1)(I-I_1)^2}{(\alpha_0+\alpha_1U_1+\alpha_2V_1+\alpha_3U_1V_1)(\rho+\delta)\,U_1} \\
& -F(U_1,V_1)V_1\left(5-\frac{F(U_1,V_1)}{F(U,V_1)}-\frac{C_1I}{CI_1}-\frac{F(U,V)}{F(U_1,V_1)}\frac{VI_1}{V_1I}-\frac{CV_1}{C_1V}-\frac{F(U,V_1)}{F(U,V)}\right) \\
& \left.+\frac{F(U_1,V_1)V_1(\alpha_0+\alpha_1U)(\alpha_2+\alpha_3U)(V-V_1)^2}{(\alpha_0+\alpha_1U+\alpha_2V_1+\alpha_3UV_1)(\alpha_0+\alpha_1U+\alpha_2V+\alpha_3UV)V_1}\right]dx \\
& -\frac{\epsilon+\rho}{\kappa}d_CC_1\int_{\Omega}\frac{\|\nabla C\|^2}{C^2}dx-\frac{(\epsilon+\rho)(\eta+\rho)}{\kappa\eta}d_VV_1\int_{\Omega}\frac{\|\nabla V\|^2}{V^2}dx.
\end{aligned}
$$

Since $5-\frac{F(U_1,V_1)}{F(U,V_1)}-\frac{C_1I}{CI_1}-\frac{F(U,V)}{F(U_1,V_1)}\frac{VI_1}{V_1I}-\frac{CV_1}{C_1V}-\frac{F(U,V_1)}{F(U,V)}\leq 0$, we have $D^{\alpha}L_1(t)\leq 0$ if $\mathcal{R}_0>1$ and $\epsilon I_1\leq\delta U_1$. The last condition is equivalent to

$$
\mathcal{R}_0\leq 1+\frac{[\delta\rho\nu(\eta+\rho)+\alpha_2\delta\sigma\kappa\eta]\,(\epsilon+\rho)+\alpha_3\epsilon\eta\kappa\sigma^2}{\epsilon\nu\rho(\epsilon+\rho)(\alpha_0\delta+\alpha_1\sigma)}.
$$

Also, $\{P_1\}$ is the largest invariant set in $\{(U,I,C,V)\mid D^{\alpha}L_1(t)=0\}$. According to LaSalle's invariance principale, we deduce that P_1 is globally asymptotically stable. This completes the proof. ■

4 Numerical simulations

In this section, we present some numerical illustrations to support the obtained analytical results.

Let Δt be the time step size, $\Omega=[x_{\min},x_{\max}]$ and $\Delta x=(x_{\max}-x_{\min})/N$ be the space step size with N is a positive integer. The grid points for the space are $x_i=x_{\min}+i\Delta x$ for $i\in\{0,...,N\}$, and for time are $t_m=m\Delta t$ for $m\in\mathbb{N}$. From Grünwald-Letnikov method [24], the Caputo fractional derivative is approximated as follows

$$
{}^{C}\partial_t^{\alpha}l\,(x_i,t_m)\approx\frac{1}{\Delta t^{\alpha}}\sum_{j=0}^{m}\beta_j^{\alpha}l\,(x_i,t_{m-j})-\tilde{l}_m, \tag{12}
$$

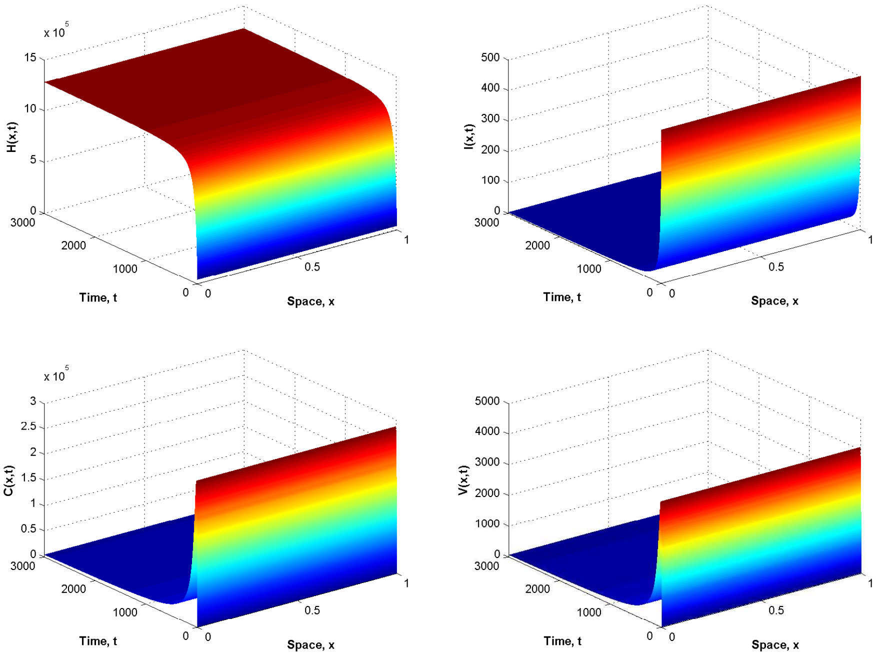

Figure 1: Stability of the infection-free steady state P_0.

where $\tilde{l}_m = \frac{l(x_i,0)t_m^{-\alpha}}{\Gamma(1-\alpha)}$ and β_j^α are the fractional binomial coefficients $\begin{pmatrix} \alpha \\ j \end{pmatrix}$ with the recursion formula

$$\beta_j^\alpha = \left(1 - \frac{1+\alpha}{j}\right)\beta_{j-1}^\alpha, \quad \beta_0^\alpha = 1.$$

Let $(U_i^m, I_i^m, C_i^m, V_i^m)$ be the approximations of the solution (U, I, C, V) of (1) at the discretized point (x_i, t_m). Then by applying (12), we get

$$\frac{1}{\Delta t^\alpha}\left(U_i^{m+1} + \sum_{j=1}^{m+1} \beta_{ji}^\alpha U^{m+1-j}\right) - \tilde{U}_i^{m+1} = \sigma - \delta U_i^m - F(U_i^m, V_i^m)V_i^m + \epsilon I_i^m,$$

$$\frac{1}{\Delta t^\alpha}\left(I_i^{m+1} + \sum_{j=1}^{m+1} \beta_j^\alpha I_i^{m+1-j}\right) - \tilde{I}_i^{m+1} = F(U_i^m, V_i^m)V_i^m - (\epsilon + \rho)I_i^m,$$

$$\frac{1}{\Delta t^\alpha}\left(C_i^{m+1} + \sum_{j=1}^{m+1} \beta_j^\alpha C_i^{m+1-j}\right) - \tilde{C}_i^{m+1} = d_C \frac{C_{i+1}^m - 2C_i^m + C_{i-1}^m}{\Delta x^2} + \kappa I_i^m - (\eta + \rho)C_i^m,$$

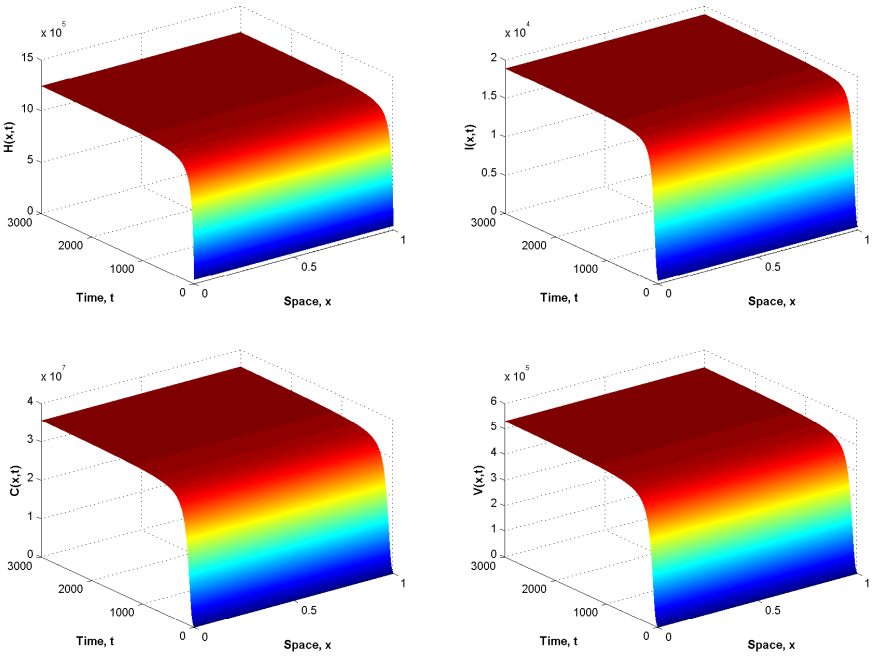

Figure 2: Stability of the chronic infection steady state P_1.

$$\frac{1}{\Delta t^{\alpha}}\left(V_i^{m+1}+\sum_{j=1}^{m+1}\beta_j^{\alpha}V_i^{m+1-j}\right)-\tilde{V}_i^{m+1}=d_V\frac{V_{i+1}^m-2V_i^m+V_{i-1}^m}{\Delta x^2}+\eta C_i^m-\nu V_i^m.$$

Hence,

$$U_i^{m+1}=-\sum_{j=1}^{m+1}\beta_j^{\alpha}U_i^{m+1-j}+\Delta t^{\alpha}\left[\tilde{U}_i^{m+1}+\sigma-\delta H_i^m-F(U_i^m,V_i^m)V_i^m+\epsilon I_i^m\right],$$

$$I_i^{m+1}=-\sum_{j=1}^{m+1}\beta_j^{\alpha}I_i^{m+1-j}+\Delta t^{\alpha}\left[\tilde{I}_i^{m+1}+F(U_i^m,V_i^m)V_i^m-(\epsilon+\rho)I_i^m\right],$$

$$C_i^{m+1}=-\sum_{j=1}^{m+1}\beta_j^{\alpha}C_i^{m+1-j}+\Delta t^{\alpha}\left[\tilde{C}_i^{m+1}+d_C\frac{C_{i+1}^m-2C_i^m+C_{i-1}^m}{\Delta x^2}+\kappa I_i^m-(\eta+\rho)C_i^m\right],$$

$$V_i^{m+1}=-\sum_{j=1}^{m+1}\beta_j^{\alpha}V_i^{m+1-j}+\Delta t^{\alpha}\left[\tilde{V}_i^{m+1}+d_V\frac{V_{i+1}^m-2V_i^m+V_{i-1}^m}{\Delta x^2}+\eta C_i^m-\nu V_i^m\right].$$

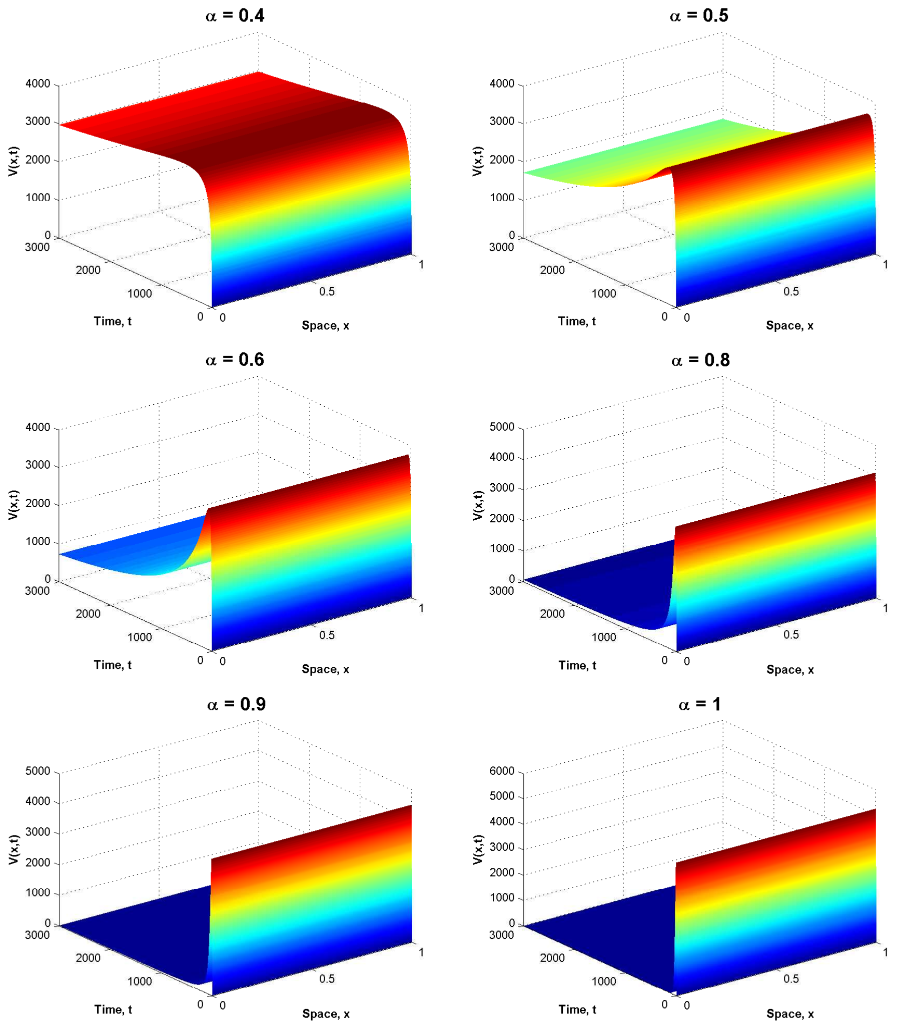

Figure 3: The state variable $V(x,t)$ with different values of α.

For numerical simulations, we choose $\Omega = [0,1]$, $d_C = 0.1$, $d_V = 0.1$, $\sigma = 50400$, $b = 3.6 \times 10^{-6}$, $\delta = 0.039$, $\rho = 0.0693$, $\kappa = 150$, $\eta = 0.01$, $\epsilon = 0.01$, $\alpha = 0.8$, $\alpha_0 = 0.1$, $\alpha_1 = 0.1$, $\alpha_2 = 0.01$ and $\alpha_3 = 0.000001$. By simple calculation, we find $\mathcal{R}_0 = 0.0128 < 1$. According to Theorem 3.1, the infection-free steady state $P_0(1.2923 \times 10^6, 0, 0, 0)$ is globally asymptotically stable which means that the virus will disappear and the patient will be completely cured. Figure 1 confirms this result.

To numerically illustrate the global stability of the second steady state of model (1), we take $b = 0.0018$ without changing the values of the other parameters. In this case, $\mathcal{R}_0 = 6.4083 > 1$. From Theorem 2.1, FPDE model (1) has the unique chronic infection steady state $P_1(1.244 \times 10^6, 1.891 \times 10^4, 3.562 \times 10^7, 5.314 \times 10^5)$. Additionally, we have

$$1 + \frac{[\delta\rho\nu(\eta+\rho) + \alpha_2\delta\sigma\kappa\eta](\epsilon+\rho) + \alpha_3\epsilon\eta\kappa\sigma^2}{\epsilon\nu\rho(\epsilon+\rho)(\alpha_0\delta+\alpha_1\sigma)} = 218.9236,$$

which implies that (11) holds. By Theorem 3.2, P_1 is globally asymptotically stable. Figure 2 validates this result.

5 Conclusions

In this article, we have presented a fractional reaction-diffusion HBV model that takes into account the HBV DNA-containing capsids and the cure of infected liver cells. The spatial diffusion is considered in capsids and virions, and the incidence of infection is described by Hattaf-Yousfi functional response that includes various forms existing in the literature. We have shown that the global dynamics of the FPDEs model is fully determined by a threshold parameter called the basic reproduction number and labeled by $\mathcal{R}_0$. More concretely, the infection-free steady state P_0 is globally asymptotically stable if $\mathcal{R}_0 \leq 1$, which biologically means that the HBV is cleared. However, the chronic infection steady state P_1 is globally asymptotically stable when $\mathcal{R}_0 > 1$ and the condition (11) holds. In this case, the HBV persists in the liver and the infection becomes chronic.

According to the above analytic results and the numerical simulations, we deduce that the diffusion and the order of fractional derivative in sense of Caputo have no effects on the stability of both steady states, but they can affect the time for arriving to these steady states. For example, the trajectories quickly converge towards the equilibria of the model for higher values of the fractional derivative order (see, Figures 3). On the other hand, the models and results presented in [8, 9, 12–16] are improved and extended.

Data Availability

The data used to support the findings of this study are available from the corresponding author upon request.

Conflicts of Interest

The authors declare that they have no conflicts of interest.

Acknowledgements

The authors would like to thank the editor and anonymous reviewers for their constructive comments and suggestions, which have improved the quality of the manuscript.

References

[1] K. Hattaf, A New Generalized Definition of Fractional Derivative with Non-Singular Kernel, Computation 8 (2) (2020), https://doi.org/10.3390/computation8020049.

[2] K. M. Owolabi, Z. Hammouch, Spatiotemporal patterns in the Belousov-Zhabotinskii reaction systems with Atangana-Baleanu fractional order derivative, Physica A 523 (2019) 1072–1090.

[3] A. Yokuş, Sema Gülbahar, Numerical Solutions with Linearization Techniques of the Fractional Harry Dym Equation, Applied Mathematics and Nonlinear Sciences 4 (1) (2019) 35–42.

[4] W. Gao, P. Veeresha, D. G. Prakasha, H. M. Baskonus, G. Yel, New Numerical Results for the Time-Fractional Phi-Four Equation Using a Novel Analytical Approach, Symmetry, 12(478) (2020) 1–16.

[5] Y. Zhang, C. Cattani, X.J. Yang, Local Fractional Homotopy Perturbation Method for Solving Non-Homogeneous Heat Conduction Equations in Fractal Domains, 17 (10) (2015) 6753–6764.

[6] W. Gao, P. Veeresha, D. G. Prakasha, H. M. Baskonus, G. Yel, New approach for the model describing the deathly disease in pregnant women using Mittag-Leffler function, Chaos, Solitons and Fractals 134 (2020) 109696

[7] WHO, Hepatitis B, July 2017. Available online: http://www.who.int/news-room/fact-sheets/detail/hepatitis-b.

[8] K. Manna and S.P. Chakrabarty, Global stability and a non-standard finite difference scheme for a diffusion driven HBV model with capsids, J. Differ. Equ. Appl. 21 (2015) 918–933.

[9] K. Manna and S. P. Chakrabarty, Chronic hepatitis B infection and HBV DNA-containing capsids: Modeling and analysis, Communications in Nonlinear Science and Numerical Simulation 22 (2015) 383–395.

[10] T.Guo, H.Liu, C.Xu, and F.Yan, Global stability of a diffusive and delayed HBV infection model with HBV DNA-containing capsids and general incidence rate. Discrete and Continuous Dynamical Systems 23 (2018) 4223–4242.

[11] K. Hattaf and N. Yousfi, Global properties of a diffusive HBV infection model with cell-to-cell transmission and three distributed delays, Disease Prevention and Health Promotion in Developing Countries, Springer, Cham, (2020) 117–131.

[12] M. Bachraou, K. Hattaf and N. Yousfi, A Fractional Order Model for HBV Infection with Capsids and Cure Rate, In: R.Mondaini (eds) Trends in Biomathematics: Mathematical Modeling for Health, Harvesting, and Population Dynamics. Springer, Cham (2019) 359–371.

[13] K.Manna and S. P. Chakrabarty, Dynamics and analysis of a model for chronic hepatitis B infection, Journal of Interdisciplinary Mathematics (2017) 339–355.

[14] X. Zhou, Q. Sun, Stability analysis of a fractional-order HBV infection model, Int. J. Adv. Appl. Math. and Mech. 2 (2) (2014) 1–6.

[15] S. M. Salman, A. M. Yousef, On a fractional-order model for HBV infection with cure of infected cells, Journal of the Egyptian Mathematical Society 25 (2017) 445–451.

[16] L. C. Cardoso, F. L. P. Dos Santos, R. F. Camargo, Analysis of fractional-order models for hepatitis B, Comp. Appl. Math. (2018) https://doi.org/10.1007/s40314-018-0588-4.

[17] K. Hattaf and N. Yousfi, A class of delayed viral infection models with general incidence rate and adaptive immune response, Int. J. Dynam. Control 4 (2016) 254–265.

[18] J. R. Beddington, Mutual interference between parasites or predators and its effect on searching efficiency, Journal of Animal Ecology 44 (1975) 331–340.

[19] D. L. DeAngelis, A. H. Goldstein, and R.V. O'Neill, A model for trophic interaction, Ecology 56 (1975) 881–892.

[20] K. Hattaf, N. Yousfi and A. Tridane, Stability analysis of a virus dynamics model with general incidence rate and two delays, Appl. Math. Comput. 221 (2013) 514–521.

[21] A. A. Arafa, S. Z. Rida, and M. Khalil, A fractional-order model of HIV infection: numerical solution and comparisons with data of patients, International Journal of Biomathematics, 7 (4) (2014) 1–11.

[22] C. V. De-Leon, Volterra-type Lyapunov functions for fractional-order epidemic systems, Communications in Nonlinear Science and Numerical Simulation 24 (2015) 75–85.

[23] J. Huo, H. Zhao, L. Zhu, The effect of vaccines on backward bifurcation in a fractional order HIV model, Nonlinear Analysis: Real World Applications 26 (2015) 289–305.

[24] I. Petras, Fractional derivatives, fractional integrals, fractional differential equations in matlab, In Engineering Education and Research Using MATLAB, InTech, 2011.
