# Supplementary material for: A Recurrence-Specific Gene-Based Prognosis Prediction Model for Lung Adenocarcinoma through Machine Learning Algorithm
Source: Biomed Res Int. 2020 Nov 7;2020:9124792. doi: 10.1155/2020/9124792 (PMC7669350; doi:10.1155/2020/9124792)
Supplement: Supplementary Materials — Figure S1: univariate Cox regression analysis to screen out the recurrence-free survival-related differentially expressed genes. Figure S2: efficiency of prediction model under different clinical subgroups. The K-M curve confirmed that the signature could significantly distinguish low- and high-risk groups in the sex subgroups (A and B), age subgroups (C and D), and location in lung parenchyma subgroups (E and F). Table S1: differential expressed genes between recurrent and primary LUAD. Table S2: differential expressed genes between high and low risks of LUAD patients of TCGA. [file 9124792.f1.zip › Supplementary figures .pdf]

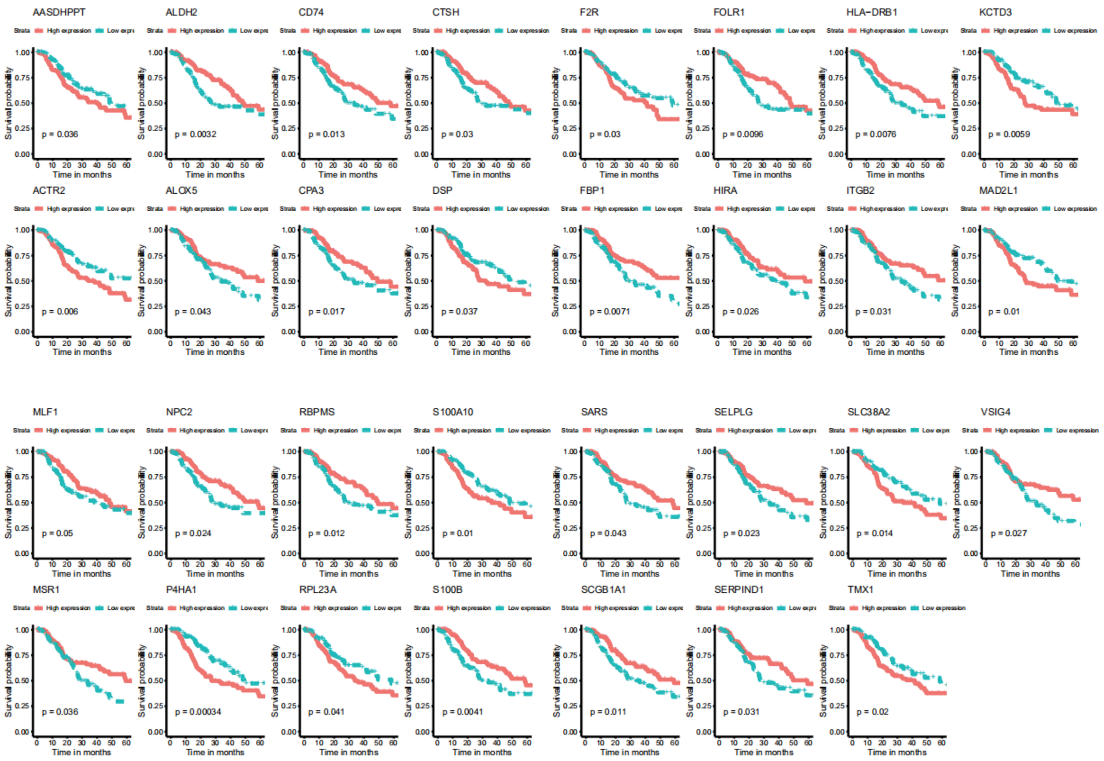

Figure S1. Univariate Cox regression analysis to screen out the recurrence-free survival-related differentially expressed genes.

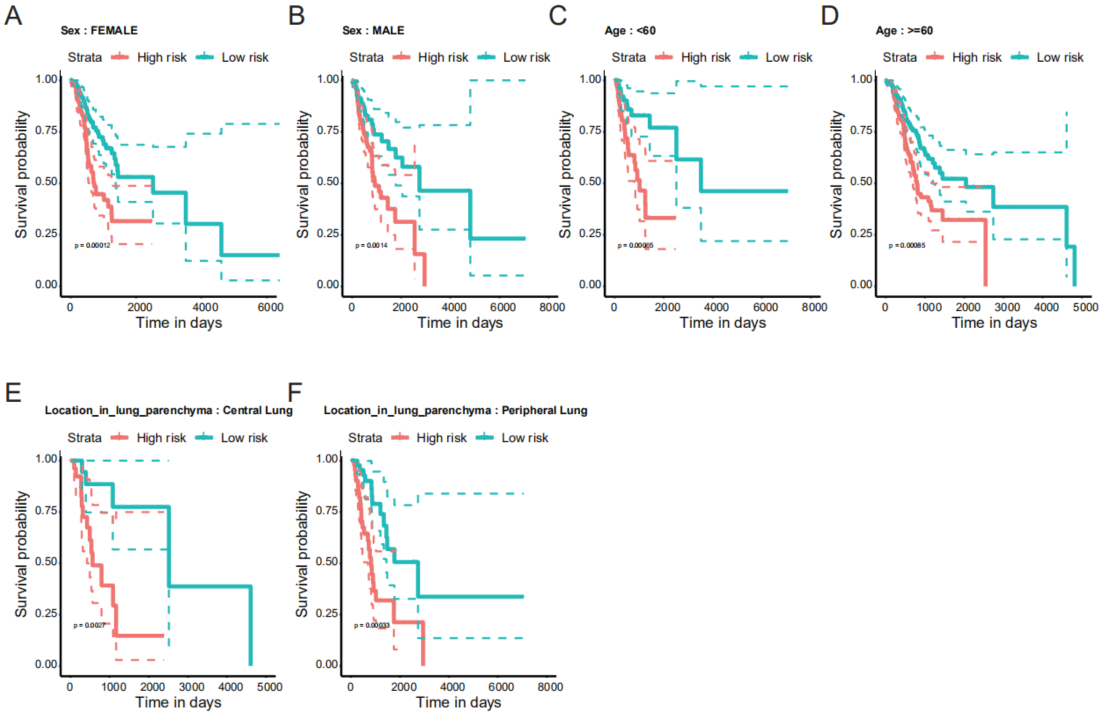

Figure S2. Efficiency of prediction model under different clinical subgroups

The K-M curve confirmed that the signature could significantly distinguish low- and high-risk groups in the sex subgroups (A and B), age subgroups (C and D) and location in lung parenchyma subgroups (E and F).
