# Supplementary material for: A Recurrence-Specific Gene-Based Prognosis Prediction Model for Lung Adenocarcinoma through Machine Learning Algorithm
Source: Biomed Res Int. 2020 Nov 7;2020:9124792. doi: 10.1155/2020/9124792 (PMC7669350; doi:10.1155/2020/9124792)
Supplement: Supplementary Materials — Figure S1: univariate Cox regression analysis to screen out the recurrence-free survival-related differentially expressed genes. Figure S2: efficiency of prediction model under different clinical subgroups. The K-M curve confirmed that the signature could significantly distinguish low- and high-risk groups in the sex subgroups (A and B), age subgroups (C and D), and location in lung parenchyma subgroups (E and F). Table S1: differential expressed genes between recurrent and primary LUAD. Table S2: differential expressed genes between high and low risks of LUAD patients of TCGA. [file 9124792.f1.zip › Table 1 (1).docx]

**Table 1. Clinical characteristics of included patients for survival model construction and validation.**

|  | **TCGA training cohort (288)** | **TCGA testing cohort (128)** | **External validation cohort (443)** |
| --- | --- | --- | --- |
| **Sex** |  |  |  |
| Female | 167 (56.04%) | 64 (50%) | 220 (49.66%) |
| Male | 131 (43.96%) | 64 (50%) | 223 (50.34%) |
| **Age** |  |  |  |
| >=60 | 201 (67.45%) | 95 (74.22%) | 315 (71.11%) |
| <60 | 88 (29.53%) | 32 (25%) | 128 (28.89%) |
| Unknown | 9 (3.02%) | 1 (0.78%) | 0 (0%) |
| **Tumor stage** |  |  |  |
| I | 171 (57.38%) | 64 (50%) | 150 (33.86%) |
| II | 69 (23.15%) | 33 (25.78%) | 252 (56.88%) |
| III | 43 (14.43%) | 21 (16.41%) | 29 (6.55%) |
| IV | 12 (4.03%) | 6 (4.69%) | 12 (2.71%) |
| Unknown | 3 (1.01%) | 4 (3.13%) | 0 (0%) |
